# Microarray data and pathway analyses for primary human activated hepatic stellate cells compared to HepG2 human hepatoma cells

**DOI:** 10.1016/j.dib.2016.12.011

**Published:** 2016-12-13

**Authors:** Alexandra M. Hetherington, Cynthia G. Sawyez, Nica M. Borradaile

**Affiliations:** Department of Physiology and Pharmacology, Schulich School of Medicine and Dentistry, Western University, London, Ontario, Canada

**Keywords:** Hepatocyte, Stellate cell, Liver, NAFLD

## Abstract

As nonalcoholic fatty liver disease progresses to end-stage diseases, including fibrosis, cirrhosis and hepatocellular carcinoma, fibrotic activated hepatic stellate cells and cancerous epithelial cells can become abundant, changing the cellular composition of this organ. Despite potentially residing within the same diseased tissue, direct comparisons of global gene expression between activated hepatic stellate cells and hepatocellular carcinoma cells are lacking. Here we provide data collected using Affymetrix GeneChip microarrays to identify differential gene expression in cultured primary human activated hepatic stellate cells compared to HepG2 human hepatoma cells. The dataset includes many genes involved in intermediary metabolism which were investigated in greater depth in our associated article (A.M. Hetherington, C.G. Sawyez, E. Zilberman, A.M. Stoianov, D.L. Robson, J.M. Hughes-Large, et al., 2016) [1]. Pathway analyses of known protein coding genes down-regulated or up-regulated by greater than 2.0-fold are also provided.

**Specifications Table**

**Table:** 

Subject area	*Hepatology*
More specific subject area	*Liver cell biology*
Type of data	*Tables, schematic images*
How data was acquired	*Affymetrix GeneChip RNA Microarray, RMA and statistical analyses*
Data format	*Filtered, analyzed*
Experimental factors	*Human activated hepatic stellate cells and HepG2 human hepatoma cells were grown to approximately 80% confluence in their respective growth media, with a change to fresh media 24 h prior to isolation of RNA.*
Experimental features	*RNA isolation, global gene expression analyses*
Data source location	*London, Ontario, Canada*
Data accessibility	*Data is within this article*

**Value of the data**

• A benchmark global gene expression analysis of primary human activated hepatic stellate cells compared to HepG2 human hepatoma cells.
• These data may be useful for comparison with microarray data from other primary liver cell types or hepatoma cell lines.
• Genes and pathways identified as differentially expressed in this data set could be investigated in future studies of the cell and molecular biology of liver diseases.

## 1 Data

**Fig. 1:**
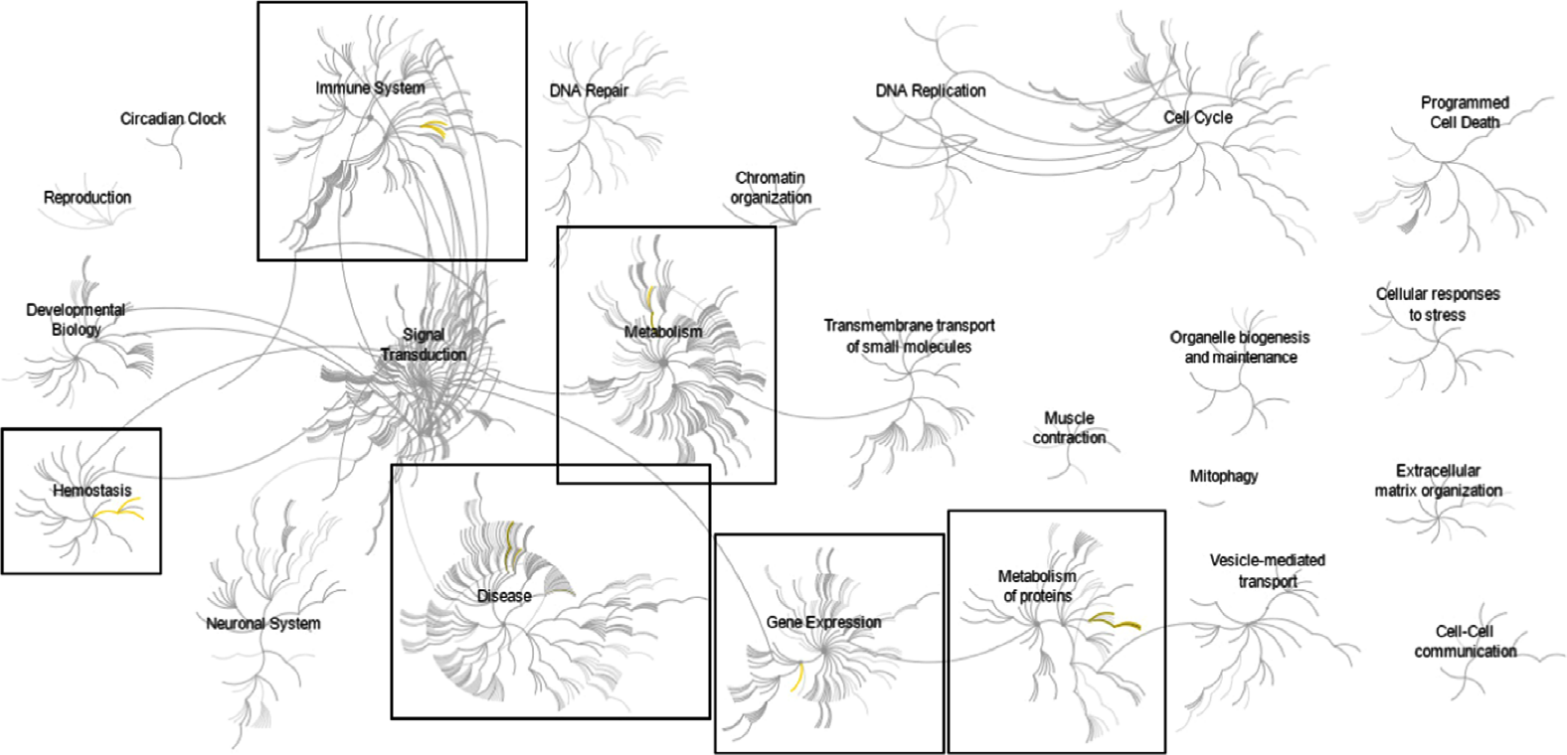
Pathways overrepresented in the list of genes down-regulated in activated hepatic stellate cells compared to HepG2 hepatoma cells. Top-level pathways are represented by central nodes, with nodes in the outer rings representing sub-pathways. Relationships between nodes are represented by arcs (edges). Significantly (*p*<0.05) overrepresented (enriched) pathways and relationships are indicated in yellow. Black boxes highlight the top-level pathways and associated sub-pathways which were identified to have significant overrepresentation. Reactome pathway identifiers, pathway names, and genes identified in significantly overrepresented pathways are provided in Supplementary material.

**Fig. 2:**
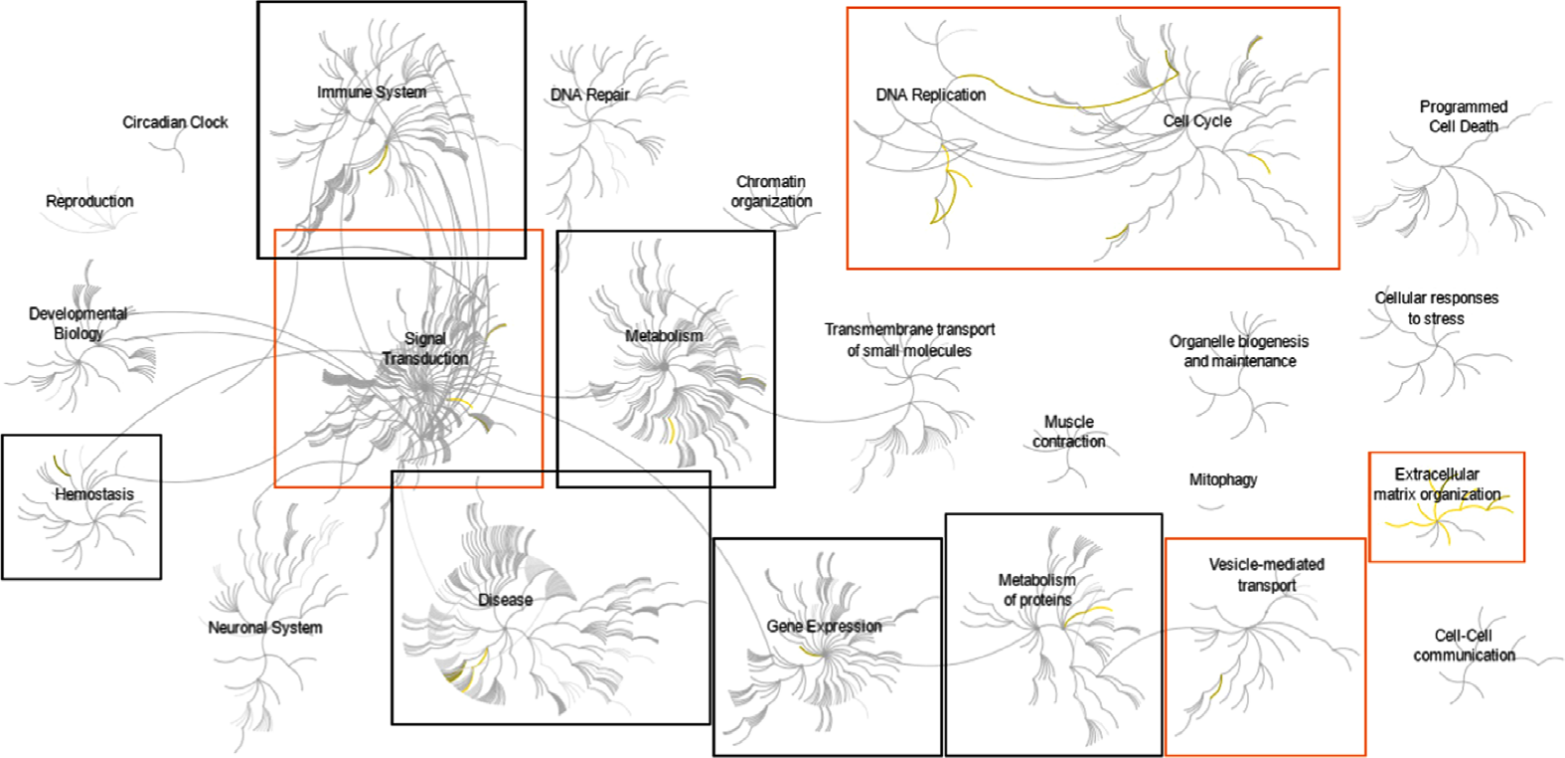
Pathways overrepresented in the list of genes up-regulated in activated hepatic stellate cells compared to HepG2 hepatoma cells. Top-level pathways are represented by central nodes, with nodes in the outer rings representing sub-pathways. Relationships between nodes are represented by arcs (edges). Significantly (*p*<0.05) overrepresented (enriched) pathways and relationships are indicated in yellow. Black boxes and orange boxes highlight the top-level pathways and associated sub-pathways which were identified to have significant overrepresentation. Orange boxes indicate top-level pathways and associated sub-pathways which were distinct from those identified in the analysis of down-regulated genes. Reactome pathway identifiers, pathway names, and genes identified in significantly overrepresented pathways are provided in Supplementary material.

Affymetrix GeneChip microarray analyses comparing mRNA isolated from primary activated human hepatic stellate cells to that of HepG2 human hepatoma cells generated a list of >6000 sequences that were differentially expressed by greater than 2.0-fold (*P*<0.01) (Supplementary material). Subsequent pathway analyses of known protein coding genes identified overrepresentation of down-regulated genes at 6 nodes representing pathways related to hemostasis, inflammation, and regulation of gene expression and metabolism (Fig. 1, and Supplementary material). Overrepresentation of up-regulated genes was identified at an additional 5 nodes including regulation of proliferation, signal transduction, vesicular transport, and regulation of extracellular matrix (Fig. 2, and Supplementary material). These data were consistent with our previous gene ontology analysis [1].

## 2 Experimental design, materials and methods

### 2.1 Cell cultures

Cryopreserved primary human activated hepatic stellate cells (HSteC) were obtained from ScienCell (Carlsbad, CA) and grown and sub-cultured according to the manufacturer׳s recommendations using their proprietary reagents, on poly-L-lysine coated culture dishes. Cells were maintained in SteCM medium containing 5.5 mM glucose, 2% fetal bovine serum (FBS), cell growth supplement (2 ng/ml each of EGF, IGF, and FGF), and penicillin/streptomycin solution (ScienCell). For experiments, cells from three independent subcultures from a single donor were used. HepG2 cells were obtained from the American Type Culture Collection (Rockville, MD) and were maintained in Eagles minimum essential medium (EMEM) (Lonza Biowhittaker) containing 5.5 mM glucose, 10% FBS, 2 mM L-glutamine, and penicillin/streptomycin solution. All cultures were incubated at 37 °C and 5% CO_2_. A total of six samples (three HSteC, three HepG2) were generated from cell monolayers at 80 percent confluence for subsequent gene expression analyses.

### 2.2 RNA Isolation, quality assessment, probe preparation and GeneChip hybridization

Total RNA was prepared as previously described [2]. Cell monolayers were harvested using trypsin and lysed with QIAshredder columns (Qiagen). Total RNA was isolated using an RNeasy Mini Kit (Qiagen), and eluted with nuclease-free water. RNA was stored at −80 °C for 1 week prior to microarray analyses.

All subsequent sample handling, labeling, and GeneChip (Human Gene 2.0 ST arrays) processing was performed at the London Regional Genomics Centre (Robarts Research Institute, London, Ontario, Canada; http://www.lrgc.ca). RNA quality was assessed using an Agilent 2100 Bioanalyzer (Agilent Technologies Inc., Palo Alto, CA) and the RNA 6000 Nano kit (Caliper Life Sciences, Mountain View, CA). Single stranded complimentary DNA was prepared from 200 ng of total RNA as per the Ambion WT Expression Kit for Affymetrix GeneChip Whole Transcript WT Expression Arrays (Applied Biosystems and Affymetrix). Total RNA was first converted to cDNA, followed by in vitro transcription to make cRNA. Single stranded cDNA was synthesized, end labeled and hybridized, for 16 h at 45 °C, to Human Gene 2.0 ST arrays (Affymetrix). All liquid handling steps were performed by a GeneChip Fluidics Station 450 and GeneChips were scanned with the GeneChip Scanner 3000 7 G (Affymetrix) using Command Console v3.2.4.

### 2.3 Statistical analyses of changes in global gene expression

All microarray data complies with MIAME guidelines. Probe level data was generated using Affymetrix Command Console v3.2.4. Probes were summarized to gene level data in Partek Genomics Suite v6.6 (Partek) using the robust multi-array average (RMA) algorithm [3]. Partek was used to determine gene level ANOVA *P*-values and fold changes. A filtered gene list was generated for expression changes of greater than 2.0-fold and having a *P*-value of less than 0.01 (Supplementary material).

### 2.4 Pathway analyses

Lists of down-regulated or up-regulated, known and predicted protein coding transcripts were identified from the complete list of >2.0-fold differentially expressed sequences. Each list was submitted for overrepresentation analyses using the open-source Reactome curated pathway database (v58) (http://www.reactome.org/). Pathway overview graphs, indicating significantly overrepresented (*p*<0.05) nodes (pathways) and relationships between nodes were generated for each list.

## Footnotes

Appendix A Supplementary data associated with this article can be found in the online version at doi:10.1016/j.dib.2016.12.011.
